# Enhanced protein fold recognition through a novel data integration approach

**DOI:** 10.1186/1471-2105-10-267

**Published:** 2009-08-26

**Authors:** Yiming Ying, Kaizhu Huang, Colin Campbell

**Affiliations:** 1 Department of Engineering Mathematics, University of Bristol, Bristol, BS8 1TR, UK; 2 National Laboratory of Pattern Recognition, Institute of Automation, The Chinese Academy of Sciences, 100190 Beijing, PR China

## Abstract

**Background:**

Protein fold recognition is a key step in protein three-dimensional (3D) structure discovery. There are multiple fold discriminatory data sources which use physicochemical and structural properties as well as further data sources derived from local sequence alignments. This raises the issue of finding the most efficient method for combining these different informative data sources and exploring their relative significance for protein fold classification. Kernel methods have been extensively used for biological data analysis. They can incorporate separate fold discriminatory features into kernel matrices which encode the similarity between samples in their respective data sources.

**Results:**

In this paper we consider the problem of integrating multiple data sources using a kernel-based approach. We propose a novel information-theoretic approach based on a Kullback-Leibler (KL) divergence between the output kernel matrix and the input kernel matrix so as to integrate heterogeneous data sources. One of the most appealing properties of this approach is that it can easily cope with multi-class classification and multi-task learning by an appropriate choice of the output kernel matrix. Based on the position of the output and input kernel matrices in the KL-divergence objective, there are two formulations which we respectively refer to as *MKLdiv-dc *and *MKLdiv-conv*. We propose to efficiently solve MKLdiv-dc by a difference of convex (DC) programming method and MKLdiv-conv by a projected gradient descent algorithm. The effectiveness of the proposed approaches is evaluated on a benchmark dataset for protein fold recognition and a yeast protein function prediction problem.

**Conclusion:**

Our proposed methods MKLdiv-dc and MKLdiv-conv are able to achieve state-of-the-art performance on the SCOP PDB-40D benchmark dataset for protein fold prediction and provide useful insights into the relative significance of informative data sources. In particular, MKLdiv-dc further improves the fold discrimination accuracy to 75.19% which is a more than 5% improvement over competitive Bayesian probabilistic and SVM margin-based kernel learning methods. Furthermore, we report a competitive performance on the yeast protein function prediction problem.

## Background

A huge number of protein coding sequences have been generated by genome sequencing projects. In contrast, there is a much slower increase in the number of known three-dimensional (3D) protein structures. Determination of a protein's 3D structure is a formidable challenge if there is no sequence similarity to proteins of known structure and thus protein structure prediction remains a core problem within computational biology.

Computational prediction of protein structure has achieved significant successes [1,2]. Focusing on the fold prediction problem of immediate interest to this paper, one computational method known as the *taxonomic *approach [3,4], presumes the number of folds is restricted and focuses on structural predictions in the context of a particular classification of 3D folds. Proteins are in a common fold if they share the same major secondary structures in the same arrangement and the same topological connections [5,6]. In the taxonomic method for protein fold classification, there are several fold discriminatory *data sources *or groups of attributes available such as amino acid composition, predicted secondary structure, and selected structural and physicochemical properties of the constituent amino acids. Previous methods for integrating these heterogeneous data sources include simply merging them together or combining trained classifiers over individual data sources [3,4,7,8]. However, how to integrate fold discriminatory data sources systematically and efficiently, without resorting to *ad hoc *ensemble learning, still remains a challenging problem.

Kernel methods [9,10] have been successfully used for data fusion in biological applications. *Kernel matrices *encode the similarity between data objects within a given input space and these data objects can include graphs and sequence strings in addition to real-valued or integer data. Thus the problem of data integration is transformed into the problem of learning the most appropriate combination of candidate kernel matrices, representing these heterogeneous data sources. The typical framework is to learn a linear combination of candidate kernels. This is often termed *multiple kernel learning *(MKL) in Machine Learning, and *non-parametric group lasso *in Statistics. Recent trends in kernel learning are usually based on the margin maximization criterion used by Support Vector Machines (SVMs) or variants [11]. The popularity of SVM margin-based kernel learning stems from its efficient optimization formulations [11-14] and sound theoretical foundation [11,15,16]. Other data integration methods include the COSSO estimate for additive models [17], kernel discriminant analysis [18], multi-label multiple kernel learning [19,20] and Bayesian probabilistic models [21,22]. These methods, in general, can combine multiple data sources to enhance biological inference [21,23] and provide insights into the significance of the different data sources used.

Following a different approach, in this paper we propose an alternative criterion for kernel matrix learning and data integration, which we will call *MKLdiv*. Specifically, we propose an information-theoretic approach to learn a linear combination of kernel matrices, encoding information from different data sources, through the use of a Kullback-Leibler divergence [24-28] between two zero-mean Gaussian distributions defined by the input matrix and output matrix. The potential advantage of this approach is that, by choosing different output matrices, the method can be easily extended to different learning tasks, such as multi-class classification and multi-task learning. These are common tasks in biological data analysis.

To illustrate the method, we will focus on learning a linear combination of candidate kernel matrices (heterogeneous data sources) using the KL-divergence criterion with a main application to the protein fold prediction problem. There are two different formulations based on the relative position of the input kernel matrix and the output kernel matrix in the KL-divergence objective. For the first formulation, although this approach involves a matrix determinant term which is not convex in general, we elegantly reformulate the learning task as a difference of convex problem, which can be efficiently solved by a sequence of convex optimizations. Hence we refer to it as *MKLdiv-dc*. The second KL-divergence formulation for kernel integration, called *MKLdiv-conv*, is convex and can be solved by a projected gradient descent algorithm. Experimental results show that these formulations lead to state-of-the-art prediction performance. In particular, MKLdiv-dc outperforms the best reported performance on the important task of protein fold recognition, for the benchmark dataset used.

## Methods

In the following we first revisit kernel learning approaches based on SVMs [11] and kernel discriminant analysis [18]. Then, we introduce our novel information-theoretic approach for data integration based on a KL-divergence criterion. Finally we discuss how to solve the optimization task efficiently. For brevity, we use the conventional notation ℕ_*n *_= {1, 2, ..., *n*} for any *n *∈ ℕ.

### Background and Related Work

Kernel methods are extensively used for biological data analysis. A symmetric function *K *: *X *× *X *→ ℝ is called *a kernel function *if it is positive semi-definite, by which we mean that, for any *n *∈ ℕ and {*x*_*i *_∈ *X*: *i *∈ ℕ_*n*_}, the *Gram matrix * is positive semi-definite. According to [29], its corresponding reproducing kernel Hilbert space (RKHS), usually denoted by ℋ_*K*_, can be defined to be the completion of the linear span of the set of functions {*K*_*x*_(·) := *K*(*x*, ·): *x *∈ *X*} with inner product satisfying, for any *x *∈ *X *and *g *∈ ℋ_*K*_, the *reproducing property *⟨*K*_*x*_, *g*⟩_*K *_= *g*(*x*). By Mercer's theorem, there exists a high dimensional (possible infinite) Hilbert feature space ℱ with inner product ⟨·, ·⟩_ℱ _and a feature map *ϕ*: *X *→ ℱ such that *K*(*x*, *t*) = ⟨*ϕ *(*x*), *ϕ *(*t*)⟩_ℱ_, ∀ *x*, *t *∈ *X*. Intuitively, the kernel function *K *implicitly maps the data space *X *into a high dimensional space ℱ, see [9,10] for more details.

Within the context of protein fold recognition, we have *m *different *fold discriminatory data sources *where *samples *across each data source can be represented by  for ℓ ∈ ℕ_*m *_and the outputs are denoted by **y **= {*y*_*i *_: *i *∈ ℕ_*n*_}. For kernel methods, for any ℓ ∈ ℕ_*m*_, each ℓ-th data source can be encoded into a candidate kernel matrix denoted by . Depending on the different data sources used, the candidate kernel function *K*_ℓ _should be specified *a priori *by the practitioner. The composite kernel matrix is given by  where {*λ*_ℓ_: ℓ ∈ ℕ_*m*_} are real-valued kernel weights and typically they are restricted to be non-negative. In this context, the problem of data integration is consequently reduced to the problem of learning a convex combination of candidate kernel matrices: more precisely learning the kernel weights *λ*. Different optimization criteria over the candidate kernels arise from the particular kernel learning algorithm used. Cristianini et al. [30] proposed a kernel learning approach which uses the cosine of the angle between the two bi-dimensional vectors **K**_*λ *_and **K_y _**representing the Gram matrices. This is achieved by maximizing the *kernel alignment*:

The above kernel learning formulation can be solved by a semi-definite programming (SDP) approach (see Section 4.7 of [11]). However, an SDP formulation is computationally intensive.

Another widely used criterion for kernel learning is based on the margin concept in SVMs and variants. Denoting the simplex set as Δ = {*λ *= (*λ*_1_, *λ*_2_, ..., *λ*_*m*_): }, Lanckriet et al [11] proposed the following formulation for kernel learning:(1)

where **1**_*n *_is a column vector of all ones, *C *is a trade-off parameter, and **t **= (*t*_1_, *t*_2_, ..., *t*_*n*_) denotes the binary outputs with *t*_*i *_∈ {1, -1} being the class label for i-th instance. This task was reformulated as a quadratically constrained quadratic programming (QCQP) problem and later improved by Sonnenburg et al. [14] who reformulated it as a semi-infinite linear programming (SILP) task. Moreover, it was pointed out in [12,13,17,31] that this is equivalent to the following sparse *L*^1^-regularization formulation:(2)

The *L*^1^-regularization term  encourages the sparsity [32] of RKHS-norm terms, and thus indicates the relative importance of data sources. It was shown in [13] that the standard *L*^2^-regularization  is equivalent to the use of uniformly weighted kernel weights *λ*, i.e.  for any ℓ ∈ ℕ_*m*_. Recently, Ye et al. [18] proposed an appealing kernel learning approach based on regularized kernel discriminant analysis. This can similarly be shown to be equivalent to a sparse *L*^1^-regularization formulation with a least square loss, see Appendix 1 for more details.

### Information-theoretic Data Integration

In this paper we adopt a novel information-theoretic approach to learn the kernel combinatorial weights. The main idea is to quantify the similarity between **K_*λ *_**and **K_y _**through a Kullback-Leibler (KL) divergence or relative entropy term [24-28]. This approach is based on noting that these kernel matrices encode the similarity of data objects within their respective input and label data spaces. Furthermore, there is a simple bijection between the set of distance measures in these data spaces and the set of zero-mean multivariate Gaussian distributions [25]. Using this bijection, the difference between two distance measures, parameterized by **K_*λ *_**and **K_y_**, can be quantified by the relative entropy or Kullback-Leibler (KL) divergence between the corresponding multivariate Gaussians. Matching kernel matrices **K_*λ *_**and **K_y _**can therefore be realized by minimizing a KL divergence between these distributions and we will exploit this approach below in the context of multiple kernel learning.

Kernel matrices are generally positive semi-definite and thus can be regarded as the covariance matrices of Gaussian distributions. As described in [24], the Kullback-Leibler (KL) divergence (relative entropy) between a Gaussian distribution (0, **K_y_**) with the output covariance matrix **K_y _**and a Gaussian distribution (0, **K**_*x*_) with the input kernel covariance matrix **K_x _**is:(3)

where, for any square matrix **B**, the notation Tr(**B**) denotes its trace. The *a priori *choice of the output matrix **K_y _**will be discussed later. Though KL ((0, **K_y_**)||(0, **K_x_**)) is non-convex w.r.t. **K_x_**, it has a unique minimum at **K_x _**= **K_y _**if **K_y _**is positive definite, suggesting that minimizing the above KL-divergence encourages **K_x _**to approach **K_y_**. If the input kernel matrix **K_x _**is represented by a linear combination of *m *candidate kernel matrices, i.e. , the above KL-divergence based kernel learning is reduced to the following formulation:(4)

where **I**_*n *_denotes the *n *× *n *identity matrix and *σ *> 0 is a supplemented small parameter to avoid the singularity of **K_*λ*_**.

Since the KL-divergence is not symmetric with respect to **K_y _**and **K_*λ*_**, another alternative approach to matching kernel matrices is given by(5)

where parameter *σ *> 0 is added to avoid the singularity of **K_y_**. If there is no positive semi-definiteness restriction over **K**_ℓ_, this formulation is a well-known convex *maximum-determinant problem *[33] which is a more general formulation than semi-definite programming (SDP), its implementation is computationally intensive, and thus cannot be extended to large-scale problems according to [33]. However, formulation (5) has a special structure here: *λ*_ℓ _is non-negative and all candidate kernel matrices are positive semi-definite. Hence, we can solve this problem by a simple projected gradient descent method, see below for more details.

The KL-divergence criterion for kernel integration was also successfully used in [27,28] which formulated the problem of supervised network inference as a kernel matrix completion problem. In terms of information geometry [34], formulation (4) corresponds to finding the *m*-projection of **K_y _**over an *e*-flat submanifold. The convex problem (5) can be regarded as finding the *e*-projection of **K_y _**over a *m*-flat submanifold. In [26], formulation (4) was developed for learning an optimal linear combination of diffusion kernels for biological networks. A gradient-based method was employed in [26] to learn a proper linear combination of diffusion kernels. This optimization method largely relies on the special property of all candidate diffusion kernel matrices enjoying the same eigenvectors and the gradient-based learning method could be a problem if we deal with general kernel matrices. In the next section, we propose to solve the general kernel learning formulation (4) using a difference of convex optimization method.

The formulation (4) also has a close relation with *Gaussian Process regression *[35]. A Gaussian process *f *can be fully specified by giving the covariance matrix for any finite set of zero-mean random variables **f **= {*f*(*x*_*i*_): *i *∈ ℕ_*m*_}. The relation between the inputs **x **= {*x*_*i *_: *i *∈ ℕ_*n*_} and outputs **y **= {*y*_*i *_: *i *∈ ℕ_*m*_} is realized by the latent variable **f **as follows:

where **I**_*n *_denotes the *n *× *n *identity matrix and the latent random variable **f **= (*f *(*x*_1_, ..., *f *(*x*_*n*_))) is distributed as a *Gaussian process prior*. The Gaussian process prior can be fully specified by a kernel *K *with a random covariance matrix  associated with random samples **x **= {*x*_*i*_: *i *∈ ℕ_*n*_}. Specifically, it can be written as **f**|**x **~(**f**| 0, **K_*λ*_**). We assume a uniform distribution over *λ*, i.e. a Dirichlet prior distribution  with *α*_0 _= 1. If we let **K_y _**= **yy**^⊤ ^in the objective function of formulation (4), then one can easily check that, up to a constant term, the objective function in formulation (4) is the negative of the log likelihood of Gaussian process regression, and maximizing the log likelihood is equivalent to the minimization problem (4).

### Optimization Formulation

We now turn our attention to optimization approaches for the KL-divergence based kernel learning formulations (4) and (5). In particular, we show that formulation (5) can be approached by a projected gradient descent method and (4) can be solved by a difference of convex algorithm (DCA) [36] which, for linear constraint conditions, reduces to the special case of a concave convex procedure (CCCP) [37]. To this end, let(6)

and(7)

**Theorem 1 ***Let functions g and f be defined by (6) and (7). Then, both f and g are convex with respect to λ *∈ Δ. *Moreover, problem (5) is convex and problem (4) is a difference of convex problem, i.e*.(8)

**Proof **It suffices to prove the convexity of *f *and *g*. To this end, from [38] we observe that functions – log |**C**| and Tr(**K_y_C**^-1^) are convex with respect to positive semi-definite matrices **C**. Hence, *f *and *g *are convex with respect to *λ *∈ Δ. This completes the proof of the theorem.

For simplicity we refer to the KL-divergence kernel learning formulation (4) as *MKLdiv-dc *since it is a difference of convex problem and refer to formulation (5) as *MKLdiv-conv *since it is a convex problem.

#### Projected Gradient Descent Method for MKLdiv-conv

We propose a projected gradient descent (PGD) method to solve problem (5). The idea of this method is to alternately implement a gradient descent and then a projection to the feasible domain, see e.g. [39]. Recall the derivative of the log determinant,(see e.g. the matrix cookbook http://matrixcookbook.com/(9)

With a little abuse of notation, we also denote by  the objective function of problem (5). Consequently, its gradient is given by(10)

Then, at iteration step *t *the gradient descent step is realized by

where *η *> 0 is a prescribed step size. The projection of *β *to the feasible domain Δ can be written as the following quadratic programming problem(11)

The theoretical convergence rate of the projected gradient descent method is generally of complexity  where *t *is the iteration number and *L *is the Lipschitz constant of the gradient function defined by (10), see e.g. [39]. Here, the Lipschitz constant *L *is bounded by the largest eigenvalue of the Hessian  of the objective function defined, for any *i*, *j *∈ ℕ_*m*_, by

Since ℒ is convex, the Hessian ℋ(ℒ) is positive semi-definite and thus(12)

where ||·||_Fro _denotes the Frobenious norm of a matrix. Hence, the projected gradient descent algorithm could take longer time to become convergent if the value of *σ *is very small.

### Difference of Convex Algorithm for MKLdiv-dc

By Theorem 1, problem (4) is a difference of convex problem. We propose to solve this problem by a concave convex procedure (CCCP) [36,37]. This procedure iteratively solves the following convex problem:(13)

where, for any *j *∈ ℕ_*m*_, the derivative of the log determinant is given by equation (9). Before we continue the main discussion, let us first note an interesting property of CCCP. By the definition of *λ*^(*t*+1)^, we know that

Since *g *is convex, we have that

Consequently,(14)

which means that the objective value ℒ(*λ*^(*t*)^) monotonically decreases with each iteration. Consequently, we can use the relative change of the objective function as a stopping criterion. Local convergence of the DCA algorithm is proven in [36] (Lemma 3.6, Theorem 3.7). Tao and An [36] state that the DCA often converges to the global solution. Overall, the DC programming approach to MKLdiv-dc can be summarized as follows.

• Given a stopping threshold *ε*

• Initialize *λ*^(1)^, e.g.  for any ℓ ∈ ℕ_*m*_

• Given the solution *λ*^(*t*) ^at step *t*, for step *t *+ 1, first compute ▽*g*(*λ*^(*t*)^) by equation (9). Then, compute solution *λ*^(*t*+1) ^of convex subproblem (13).

• Stop until the relative change  where *ε *is a stopping threshold

#### SILP Formulation for the Convex Subproblem (13)

We now turn to the solution of the convex subproblem (13). To see this, first decompose the output matrix **K_y _**into the form **K_y _**= AA^⊤^, e.g. by eigen-decomposition. Here, *A *is an *n *× *r *matrix with *r *= *rank*(*A*) which always exists since **K_y _**is positive semi-definite. Hence, by introducing an auxiliary matrix *α *∈ ℝ^*n *× *r*^, we observe, for any positive definite matrix **C**, that

Applying the above equality with , up to a constant, equation (13) is equivalent to the augmented problem:

Equivalently, by the min-max theorem (see e.g. [38])(15)

To solve the subproblem (15), we can formulate it as a quadratically constrained quadratic programming (QCQP) problem as in [11]. Here we formulate the problem in (15) as a semi-infinite linear programming (SILP) problem [14,40] since SILP usually has better scalability compared to QCQP. To see this, let , and . Then, letting , we can rewrite (15) as a SILP problem:(16)

In (16), there are an infinite number of constraints (indexed by *a*), indicative of a *semi-infinite linear programming *(SILP) problem. The SILP task can be solved by an iterative *column generation *algorithm (or exchange method) which is guaranteed to converge to a global optimum. A brief description of the column generation method is illustrated in Appedix 2.

Alternatively we could apply the projected gradient descent (PGD) method in the above subsection directly to the convex subproblem (13). However, the gradient function of its objective function involves the matrix . In analogy to the argument of inequality (12), the Lipschitz constant of the gradient of the objective function in (13) is very large when the value of *σ *is very small, and thus the projected gradient descent algorithm could take longer to become convergent. Hence, this could make the overall DC programming unacceptable slow. In contrast, in the SILP formulation (16) we introduce the auxiliary variables *α *to avoid the matrix . In addition, the gradient descent algorithm generally needs to determine the step size *η *according to the value of *σ*, see also discussion in the experimental section.

### Prior Choice of the Output Kernel Matrix

The choice of the output kernel matrix **K_y _**will depend on the problem considered. We first consider a multi-class classification for the specific task of protein fold recognition. In this case, we preprocess the output labels using a one-against-all strategy. In particular, for a *C*-class classification we recast the outputs **y **= {*y*_*i *_: *i *∈ ℕ_*n*_} as (*y*_*i*1_, ..., *y*_*iC*_) such that *y*_*ip *_= 1 if *x*_*i *_is in class *p *and otherwise -1. Hence the outputs are represented by an *n *× *C indicator matrix ***Y **= (*y*_*ip*_)_*i*, *p *_whose *p*-th column vector is denoted by **y**_*p*_. Then, taking **K_y _**= **YY**^⊤^, formulation (4) can be extended to the joint optimization problem(17)

and formulation (5) can be written as(18)

For the protein fold recognition and yeast protein function prediction projects discussed below, we choose **K_y _**= **YY**^⊤ ^as stated.

In general, though, **K_y _**might encode a known structural relationship between labels. For example, in supervised gene or protein network inference (see e.g. [41,42]) the output information corresponds to an adjacency (square) matrix *A *where *A*_*ij *_= 1 means there is an interaction between gene or protein pair (*e*_*i*_, *e*_*j*_) of an organism, otherwise *A*_*ij *_= 0. In this case, the output kernel matrix **K_y _**can potentially be chosen as the graph Laplacian defined as *L *= diag(*A***1**) - *A*, where **1 **is the vector of all ones. It can also be formulated as a diffusion kernel [43] defined by , where hyper-parameter *β *> 0. Other potential choices of **K_y _**can be found in [19,20] for multi-labeled datasets.

## Results and Discussion

We mainly evaluate MKLdiv methods (**MKLdiv-dc **and **MKLdiv-conv**) on protein fold recognition, and then consider an extension to the problem of yeast protein function prediction. In these tasks we first compute the kernel weights by MKLdiv and then feed these into a one-against-all multi-class SVM to make predictions. The trade-off parameter in the multi-class SVM is adjusted by 3-fold cross validation over the training dataset. For all experiments with MKLdiv-dc, we choose *σ *= 10^-5 ^and for MKLdiv-conv, we tune *σ *= {10^-5^, ..., 10^-1^} using cross validation. In both methods, we use a stopping criterion of *ε *= 10^-5 ^and initialize the kernel weight *λ *by setting  for any ℓ ∈ ℕ_*m *_where *m *is the number of candidate kernel matrices.

### Synthetic Data

**Figure 1:**
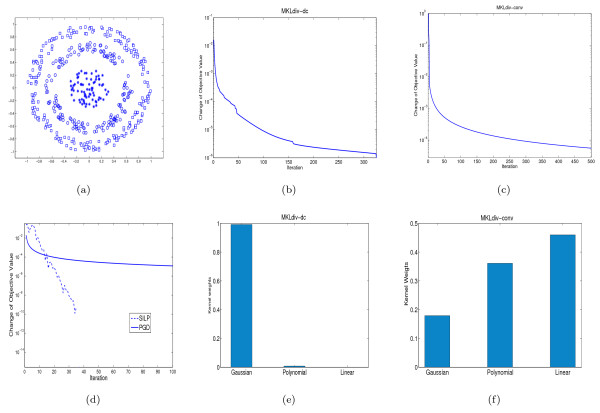
**Synthetic data verification**. Synthetic data verification of MKLdiv: (a) depiction of the three-circle dataset; (b) relative change of objective values of MKLdiv-dc versus iteration number of CCCP; (c) relative change of objective values of MKLdiv-conv versus iteration number of projected gradient descent (PGD) method; (d) relative change of objective values of subproblem (13) by SILP (dish-line) and PGD methods; (e) kernel weights learned by MKLdiv-dc; (f) kernel weights learned by MKLdiv-conv.

We first validated the proposed MKLdiv algorithms on a simple three-class dataset illustrated in subfigure (a) of Figure 1. As in [11], we use a Gaussian kernel with unit variance, a polynomial kernel of order two and a linear kernel. In this case we demonstrate the effect of our approaches on combining kernel matrices derived from a single data source. Subfigures (e) and (f) of Figure 1 illustrate the kernel weights learned by MKLdiv-dc and MKLdiv-conv. In particular, MKLdiv-dc successfully picked up the Gaussian kernel as the most dominant kernel, which is more reasonable than MKLdiv-conv. Subfigures (b) and (c) of Figure 1 show the relative change of objective function values versus iteration, i.e. (ℒ(*λ*^(*t*-1)^) - ℒ(*λ*^(*t*)^))/ℒ(*λ*^(*t*)^), of MKLdiv-dc and MKLdiv-conv. We can see that the DC algorithm of MKLdiv-dc converges quickly to a local minimum while the projected gradient descent algorithm converges a little slower to a global minimum. However, MKLdiv-dc needs more time per iteration than MKLdiv-conv since MKLdiv-dc needs to solve the subproblem (13) at each iteration. As mentioned before, the subproblem (13) can be solved by either semi-infinite linear programming (SILP) or a projected gradient descent (PGD) method. To see their convergence, in subfigure (d) of Figure 1 we plot the relative changes of the objective function in subproblem (13) when  for ℓ ∈ ℕ_*m*_. We can see from subfigure (d) that the PGD approach converges faster in the beginning but stalls at a higher precision and the SILP method converges faster at higher precision.

### Protein Fold Recognition

Next we evaluated MKLdiv on a well-known protein fold prediction dataset [3]. This benchmark dataset (based on SCOP PDB-40D) has 27 SCOP fold classes with 311 proteins for training and 383 for testing. This dataset was originally proposed by Ding and Dubchak [3] and it has 313 samples for training and 385 for testing. There is less than 35% sequence identity between any two proteins in the training and test set. We follow Shen and Chou [4] who proposed to exclude two proteins from the training and test datasets due to a lack of sequence information. We compare our MKLdiv methods with kernel learning based on one-against-all multiclass SVM using the SimpleMKL software package [44], kernel learning for regularized discriminant analysis (MKL-RKDA) [18]http://www.public.asu.edu/~jye02/Software/DKL/ and a probabilistic Bayesian model for kernel learning (VBKC) [21]. The trade-off parameters in SimpleMKL and MKL-RKDA were also adjusted by 3-fold cross validation on the training set.

#### Description of the Fold Discriminatory Data Sources

As listed in Table 1, there are a total of 12 different fold discriminatory data sources available: Amino Acid Composition (C), Predicted Secondary Structure (S), Hydrophobicity (H), Polarity (P), van der Waals volume (V), Polarizability (Z), PseAA *λ *= 1 (L1), PseAA *λ *= 4 (L4), PseAA *λ *= 14 (L14), PseAA *λ *= 30 (L30), SW with BLOSUM62 (SW1) and SW with PAM50 (SW2). The first six data sources were originally from [3]. Four data sources using different dimensions of pseudo-amino acid composition (PseAA) were introduced in [4] to replace the amino-acid composition. The last two data sources used in [21] are derived from a pairwise kernel [45] for local sequence alignment based on Smith-Waterman scores.

**Table 1:** Performance with individual and all data sources

Data sources	MKLdiv-dc	MKLdiv-conv	SimpleMKL	VBKC	MKL-RKDA
Amino acid composition (C)	51.69	51.69	51.83	51.2 ± 0.5	45.43
Predicted secondary structure (S)	40.99	40.99	40.73	38.1 ± 0.3	38.64
Hypdrophobicity (H)	36.55	36.55	36.55	32.5 ± 0.4	34.20
Polarity (P)	35.50	35.50	35.50	32.2 ± 0.3	30.54
van der Walls volume (V)	37.07	37.07	37.85	32.8 ± 0.3	30.54
Polarizability (Z)	37.33	37.33	36.81	33.2 ± 0.4	30.28
PseAA *λ *= 1 (L1)	44.64	44.64	45.16	41.5 ± 0.5	36.55
PseAA *λ *= 4 (L4)	44.90	44.90	44.90	41.5 ± 0.4	38.12
PseAA *λ *= 14 (L14)	43.34	43.34	43.34	38 ± 0.2	40.99
PseAA *λ *= 30 (L30)	31.59	31.59	31.59	32 ± 0.2	36.03
SW with BLOSUM62 (SW1)	62.92	62.92	62.40	59.8 ± 1.9	61.87
SW with PAM50 (SW2)	63.96	63.96	63.44	49 ± 0.7	64.49

All data sources	**73.36**	**71.01**	66.57	**68.1 ± 1.2**	**68.40**
Uniform weighted	68.40	68.40	**68.14**	-	66.06

The results of VBKC are cited from [21]. The results not employed there are denoted by '-'. The best result for each kernel learning method is marked in bold.

As in [21], we employ linear kernels (Smith-Waterman scores) for SW1 and SW2 and second order polynomial kernels for the other data sources. Ding and Dubchak [3] conducted an extensive study on the use of various multi-class variants of standard SVMs and neural network classifiers. For these authors the best test set accuracy (TSA) was 56%, and the most informative among their six data sources (CSHPVZ) were amino-acid composition (C), the predicted secondary structure (S) and hydrophobicity (H). Shen and Chou [4] introduced four additional PSeAA data sources to replace the amino acid composition (C) and raised test performance to 62.1%. The latter authors used an *ad hoc *ensemble learning approach involving a combination of multi-class *k *nearest neighbor classifiers individually trained on each data source. Recently, test performance was greatly improved by Damoulas and Girolami [21] using a Bayesian multi-class multi-kernel algorithm. They reported a best test accuracy of 70% on a single run.

#### Performance with Individual and All Data Sources

We ran MKLdiv-dc, MKLdiv-conv, SimpleMKL and MKL-RKDA on the overall set of 12 data sources, also evaluating performance on a uniformly weighted (averaged) composite kernel in addition to individual performance on each separate data source. In Table 1 we report the test set accuracy on each individual data source. The performance of MKLdiv-dc and MKLdiv-conv inclusive of all data sources achieves a test set accuracy of 73.36% and 71.01% respectively, consistently outperforming all individual performances and the uniformly weighted composite kernel (68.40%). Moreover, individual performance for MKLdiv-dc, SimpleMKL and MKL-RKDA indicates that the most informative data sources are local sequence alignments (SW1 and SW2) and the amino acid composition (C). The performance with individual data sources for MKLdiv-dc, MKLdiv-conv, and SimpleMKL are almost the same since, for a fixed kernel, they use the same one-against-all multi-class SVM.

**Figure 2:**
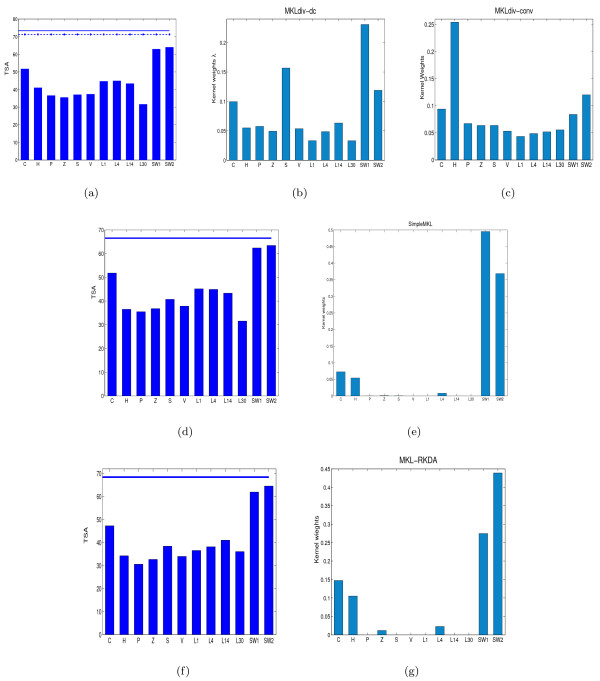
**Performance with all data sources on protein fold recognition**. Test set accuracy of individual (bars) and all data sources (horizontal lines) on the protein fold recognition dataset: (a) MKLdiv-dc and MKLdiv-conv, where the solid line is the performance of MKLdiv-dc and the star-dashed line is the performance of MKLdiv-conv; (d) SimpleMKL; (f) MKL-RKDA. Kernel weights: (b) MKLdiv-dc, (c) MKLdiv-conv, (e) SimpleMKL and (g) MKL-RKDA.

From Table 1, performances of MKLdiv-dc and MKLdiv-conv with all the available data sources achieve test set accuracies of 73.36% and 71.01%, both of which outperform the state-of-art performance 70% on a single run reported in [21] and other kernel learning methods including SimpleMKL (66.57%) and MKL-RKDA (68.40%). The performance of the uniformly weighted kernel is 68.14% which is better than the performance 66.57% of SimpleMKL. This indicates that sparse *L*^1^-regularization does not necessarily yield better performance. The kernel weights *λ *of MKLdiv-dc, SimpleMKL, and MKL-RKDA are shown in subfigures (b), (e) and (g) of Figure 2 which indicates that Amino Acid Composition (C), predicted secondary structure (S), Hypdrophobicity (H), and the last two data sources SW1 and SW2 are the most informative data sources, and the remaining data sources of H, P, V, and PseAA are less informative. As depicted in the subfigure (b) of Figure 2, MKLdiv-dc and MKLdiv-conv include some less informative data sources such as P, Z, L1, L4, L14, L30 etc., with small (but not zero) kernel weights. In contrast, as shown in (e) and (g) of Figure 2, SimpleMKL and MKL-RKDA completely discard these less informative data sources. However, as shown in (d) and (f) of Figure 2, SimpleMKL and MKL-RKDA achieve poorer performance, less than 70%, while MKLdiv-dc achieves 73.36% and MKLdiv-conv achieves 71.01%. This suggests that MKLdiv-dc provides a more reasonable balance over the entire set of data sources. This observation also suggests that achieving a sparsity among kernel weights does not necessarily guarantee good generalization performance since some available data sources may be weakly informative but may still carry some useful additional information.

#### Performance with Sequential Addition of Data Sources

As mentioned above, the kernel weights learned by MKLdiv on all the data sources can provide useful insights into the significance of informative data sources. Hence, we further investigated the effect of sequentially adding data sources based on information from learned kernel weights in Tables 2 and 3. Without loss of generality, we take the kernel weights learned by MKLdiv-dc as an example.

**Table 2:** Effects of sequentially adding data sources

Data sources	MKLdiv-dc	MKLdiv-conv	VBKC	SimpleMKL	MKL-RKDA
C	51.69	51.69	51.2 ± 0.5	51.69	47.25

CS	56.39 (20.23 *s*)	55.35 (0.32 *s*)	55.7 ± 0.5 (-)	55.61 (14.67 *s*)	48.30 (0.15 *s*)

CSH	57.70 (50.35 *s*)	58.22 (2.44 *s*)	57.7 ± 0.6 (-)	56.91 (10.40 *s*)	55.61 (0.12 *s*)

CSHP	58.48 (39.02 *s*)	53.52 (72.14 *s*)	57.9 ± 0.9 (-)	57.96 (17.84 *s*)	56.65 (0.18 *s*)

CSHPV	60.05 (75.05 *s*)	53.26 (86.39 *s*)	58.1 ± 0.8 (-)	57.96 (15.05 *s*)	55.87 (0.17 *s*)

CSHPVZ	59.26 (135.08 *s*)	53.52 (99.64 *s*)	58.6 ± 1.1 (-)	59.00 (20.02 *s*)	57.70 (0.20 *s*)

CSHPVZL1	60.05 (221.75 *s*)	52.74 (122.74 *s*)	60.0 ± 0.8 (-)	61.35 (27.38 *s*)	57.70 (0.21 *s*)

CSHPVZL1L4	62.14 (315.70 *s*)	52.74 (129.08 *s*)	60.8 ± 1.1 (-)	61.61 (151.38 *s*)	58.22 (0.25 *s*)

CSHPVZL1L4L14	62.14 (450.57 *s*)	61.09 (57.09 *s*)	61.5 ± 1.2 (-)	60.05 (42.81 *s*)	59.53 (0.25 *s*)

CSHPVZL1L4L14L30	62.14 (612.72 *s*)	62.14 (67.29 *s*)	62.2 ± 1.3 (-)	62.40 (64.74 *s*)	55.61 (0.25 *s*)

CSHPVZL1L4L14L30SW1	71.80 (620.16 *s*)	**71.54** (17.97 *s*)	66.4 ± 0.8 (-)	65.79 (78.94 *s*)	66.84 (0.31 *s*)

CSHPVZL1L4L14L30SW1SW2	**73.36** (805.11 *s*)	71.01 (84.21 *s*)	**68.1 ± 1.2** (-)	**66.57** (196.42 *s*)	**68.40** (0.31 *s*)

SHPVZL1L4L14L30	60.57 (438.89 *s*)	61.09 (67.92 *s*)	61.1 ± 1.4 (-)	59.00 (44.79 *s*)	54.56 (0.25 *s*)

The result of Bayesian kernel learning model (VBKC) is cited from [21]. The results not employed there are denoted by '-'. The term inside the parenthesis is the CPU running time (seconds). The best test set accuracy of each kernel learning method is marked in bold.

**Table 3:** Effects of sequentially adding data sources (continued)

Data sources	MKLdiv-dc	MKLdiv-conv	SimpleMKL	MKL-RKDA
SW1	62.92	62.92	62.40	61.87

SW1S	65.27 (24.72 *s*)	66.31 (10.49 *s*)	64.22 (40.60 *s*)	64.75 (0.12 *s*)

SW1SW2S	67.10 (48.79 *s*)	66.05 (4.65 *s*)	64.75 (61.71 *s*)	64.49 (0.15 *s*)

SW1SW2CS	73.36 (40.65 *s*)	72.32 (23.43 *s*)	65.01 (62.81 *s*)	67.62 (0.17 *s*)

SW1SW2CSH	74.67 (72.19 *s*)	72.32 (8.69 *s*)	66.31 (75.11 *s*)	67.88 (0.15 *s*)

SW1SW2CSHP	74.93 (123.98 *s*)	**74.41** (11.63 *s*)	66.31 (74.85 *s*)	**69.71** (0.18 *s*)

SW1SW2CSHPZ	**75.19** (189.91 *s*)	73.36 (15.00 *s*)	**68.92** (109.09 *s*)	66.05 (0.20 *s*)

SW1SW2CSHPZV	74.41 (278.47 *s*)	**74.41** (17.47 *s*)	66.31 (117.14 *s*)	69.19 (0.25 *s*)

SW1SW2CSHPZVL1	73.10 (404.82 *s*)	73.32 (49.41 *s*)	66.84 (101.01 *s*)	68.66 (0.25 *s*)

SW1SW2CSHPZVL1L4	72.84 (576.29 *s*)	72.06 (57.83 *s*)	67.10 (107.88 *s*)	67.62 (0.25 *s*)

SW1SW2CSHPZVL1L4L14	72.58 (748.72 *s*)	72.36 (19.43 *s*)	66.84 (163.85 *s*)	69.19 (0.28 *s*)

SW1SW2CSHPZVL1L4L14L30	73.36 (811.54 *s*)	71.01 (83.93 *s*)	66.57 (197.57 *s*)	68.40 (0.31 *s*)

Test set accuracy of sequentially adding fold discriminatory data sources (continued) according to the ranking of kernel weights obtained by MKLdiv-dc over all data sources. The results of the Bayesian kernel learning method were not employed in [21], hence we do not list in the table. The term inside the parenthesis is the CPU running time (seconds). The best test set accuracy of each kernel learning method is marked in bold.

We first report in Table 2 the effect of sequentially adding the sources in the order which was used in [3] and [21] and MKLdiv-dc and MKLdiv-conv consistently outperform the competitive kernel learning methods VBKC, SimpleMKL, MKL-RKDA and the best performing SVM combination methodology stated in [3]. As suggested by the kernel weights of MKLdiv-dc in the subfigure (b) of Figure 2, the sequence alignment based data source SW1 is most informative, then S, then SW2 and so on. Hence, in Table 3 we further report the effect of sequentially adding data sources in this rank order. As shown in Table 3, there is a significant improvement over SW1SW2 in MKLdiv-dc when we sequentially add the data sources of amino acid composition (C) and predicted secondary structure (S). The performance of MKLdiv-dc keeps increasing until we include CSHPZ, giving the best performance of 75.19%. Although according to [4], the PseAA data sources are believed to contain more information than the conventional amino acid composition. The same behaviour appears for MKLdiv-conv. However, the MKLdiv-dc performance degenerates if we continue to add PseAA composition data sources and the same behaviour appears for MKLdiv-conv. Similar observations were made by [21] which suggests that PseAA measurements may carry non-complementary information with the conventional amino acid compositions.

**Figure 3:**
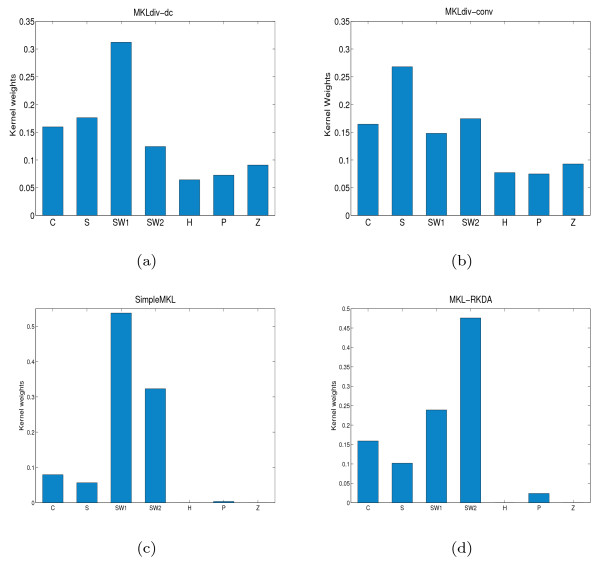
**Kernel weights on dominant data sources for protein fold recognition**. Kernel weights on the dominant data sources SW1SW2CSHPZ which yields the best prediction on the protein fold recognition dataset: (a) MKLdiv-dc, (b) MKLdiv-conv, (c) SimpleMKL and (d) MKL-RKDA.

With regard to the best performance of MKLdiv-dc with the feature set SW1SW2CSHPZ, we display the corresponding kernel weights in Figure 3. We can see in Figure 3 that SimpleMKL and MKL-RKDA almost eliminate the informative feature set HPZ while MKLdiv-dc and MKLdiv-conv include them into the composite kernel. The sparse *L*^1^-regularization of SimpleMKL and MKL-RKDA accounts for the sparse weights of SimpleMKL and MKL-RKDA.

#### Comparison of Running Time

To investigate the run-time efficiency of MKLdiv on protein fold recognition dataset, we list their CPU time in Tables 2 and 3. The running time (in seconds) is the term inside the parenthesis. The SILP approach for MKL-RKDA is very efficient while SimpleMKL takes a bit longer. The reason could be that MKL-RKDA essentially used the least-square loss for multi-class classification in contrast to the one-against-all SVM used in SimpleMKL. Generally, more time is required to run the interior method for one-against-all SVM than directly computing the solution of the least-square regression. The projected gradient descent method for MKLdiv-conv is also slower than MKL-RKDA. It is to be expected that MKLdiv-conv converges faster than MKLdiv-dc since the DC algorithm for MKLdiv-dc is non-convex and it needs to solve the subproblem (13) in each iteration of CCCP. Nevertheless, the price we paid in running time for MKLdiv-dc is worthwhile given its significantly better performance on the protein fold prediction problem.

#### Sensitivity against Parameter *σ*

**Figure 4:**
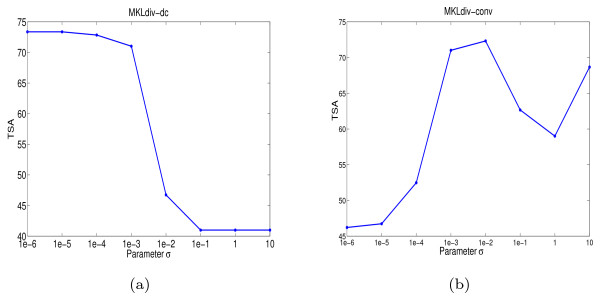
**Sensitivity against parameter *σ *for protein fold recognition**. Test set accuracy versus different values of *σ *on the protein fold recognition dataset: (a) MKLdiv-dc and (b) MKLdiv-conv.

The initial purpose of introducing *σ *is to avoid the singularity of the input kernel matrix or the output kernel matrix. However, in practice we found that, in the convex formulation MKLdiv-conv, values of *σ *have a great influence on performance for protein fold recognition. Hence, when we ran MKLdiv-conv, we always did cross validation over the training set to select the parameter σ. To see how sensitive the test set accuracy is with respect to σ, in Figure 4 we depicted the test set accuracy versus values of σ. In Figure 4 we can observe that the test set accuracy of MKLdiv-dc is relatively stable for small values of *σ*'s. However, this is not the case for MKLdiv-conv and generally suggests that the parameter *σ *has a great impact on performance of MKLdiv-conv. This could be because the output kernel matrix **K_y _**= **YY**^⊤ ^is of low rank (rank one in binary classification) and thus adding a small matrix *σ***I**_*n *_in the formulation MKLdiv-conv could dramatically change the information of the output kernel matrix. In contrast, we can reasonably assume the input kernel matrices are non-singular or not of low rank and the effect of adding a small matrix *σ***I**_*n *_in the formulation MKLdiv-dc can be ignored.

### Extension of Investigation to Yeast Protein Classification

We next extend our investigation of MKLdiv-dc and MKLdiv-conv on a yeast membrane protein classification problem [23]. This binary classification task has 2316 examples derived from the MIPS comprehensive Yeast Genome Database (CYGD) (see [46]). There are eight kernel matrices http://noble.gs.washington.edu/proj/sdp-svm/. The first three kernels (*K*_SW_, *K*_B_, and *K*_Pfam_) are respectively designed to measure the similarity of protein sequences using BLAST, Smith-Waterman pairwise sequence comparison algorithms and a generalization of pairwise comparison method derived from hidden Markov models. The fourth sequence-based kernel matrix (*K*_FFT_) incorporates information about hydrophobicity which is known to be useful in identifying membrane proteins, computed by Fast Fourier Transform. The fifth and sixth kernel matrices (*K*_LI_, *K*_D_) are respectively derived from linear and diffusion kernels based on protein-protein interaction information. The seventh kernel matrix (*K*_E_) is a Gaussian kernel encoding gene expression data. Finally, we added a noise kernel matrix *K*_Ran _generated by first generating random numbers and then using a linear kernel.

**Figure 5:**
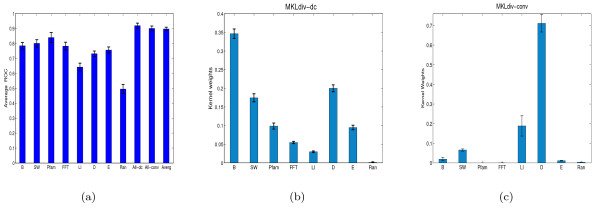
**Performance of MKLdiv on yeast membrane protein**. Performance on the yeast membrane protein function dataset: (a) average ROC score for individual data sources, using MKLdiv-dc and MKLdiv-conv, where the third bar to last (All-dc) is MKLdiv-dc, the second bar to last (All-conv) is MKLdiv-conv and the last bar (Averg) is the performance using uniformly weighted kernels. Kernel weights: (b) MKLdiv-dc and (c) MKLdiv-conv.

The performance of MKLdiv-dc and MKLdiv-conv is evaluated by 10 random partitions of the data into a training and test set in a proportion of 4: 1. We report the receiver operating characteristic (ROC) score, which measures the overall quality of the ranking induced by the classifier, rather than the quality of a single point in that ranking. The first subfigure of Figure 5 shows the performance with individual kernels and the performance of MKLdiv-dc (the third to last bar), MKLdiv-conv (the next to last bar), and the uniformly weighted kernel (last bar). Specifically, MKLdiv-dc yields a ROC score of 0.9189 ± 0.0171 which is competitive with the result in [23]. MKLdiv-conv, however, achieved a ROC score of 0.9016 ± 0.0161 which is worse than MKLdiv-dc. The performance of MKLdiv-dc is also slightly better than the performance of the uniformly weighted kernel 0.9084 ± 0.0177 excluding the noise kernel and 0.8979 ± 0.0120 including the noise kernel. We also plot the kernel weights on (b) and (c) of Figure 5. As expected, in MKLdiv-dc the BLAST kernel (*K*_*B*_) derived from the protein sequence similarity comparison is very informative which is consistent with [23]. The derived kernel weights also show that the interaction-based diffusion kernel is more informative than the expression kernel, which is consistent with [23]. Also, it is interesting to note that MKLdiv-dc shows that the noise kernel (*K*_Ran_) is least informative. This is indicated by its individual ROC score: a ROC score around 0.5 corresponds to random ranking. The kernel weights of MKLdiv-conv indicate that the diffusion kernel (D) is the most important data source, and also suggest that Pfam and FFT are almost non-informative regardless of their good individual performances. For the kernel weights, MKLdiv-dc is more reasonable than MKLdiv-conv since MKLdiv-dc is more consistent with the individual data source's performance and MKLdiv-dc outperforms MKLdiv-conv using all data sources.

## Conclusion

In this paper we developed a novel information-theoretic approach to learning a linear combination of kernel matrices based on the KL-divergence [24-28], especially focused on the protein fold recognition problem. Based on the different position of the input kernel matrix and the output kernel matrix in the KL-divergence objective, there are two formulations. The first one is a difference of convex (DC) problem termed MKLdiv-dc and the second formulation is a convex formulation called MKLdiv-conv. The sparse formulation for kernel learning based on discriminant analysis [18] was also established. Our proposed methods are able to achieve state-of-the-art performance on the SCOP PDB-40D benchmark dataset for protein fold recognition problem. In particular, MKLdiv-dc further improves the fold discrimination accuracy to 75.19% which is a more than 5% improvement over a competitive Bayesian probabilistic approach [21], SVM margin-based kernel learning methods [11], and the kernel learning based on discriminant analysis [18]. We further extended the investigation to the problem of yeast protein function prediction.

Generally, it is difficult to determine which criterion is better for multiple kernel combination since this problem is highly data-dependent. For the information-theoretic approaches MKLdiv-dc and MKLdiv-conv, although MKLdiv-dc is not convex and its DC algorithm tends to find a local minima, in practice we would recommend MKLdiv-dc for the following reasons. Firstly, as mentioned above MKLdiv-dc has a close relation with the kernel matrix completion problem using information geometry [27,28] and the maximization of the log likelihood of Gaussian Process regression [35], which partly explains the success of MKLdiv-dc. Secondly, we empirically observed that MKLdiv-dc outperforms MKLdiv-conv in protein fold recognition and yeast protein function prediction. Finally, as we showed in Figure 4, the performance of MKLdiv-conv is quite sensitive to the parameter *σ *and the choice of *σ *remains a challenging problem. MKLdiv-dc is relatively stable with respect to small values of *σ *and we can fix *σ *to be a very small number e.g. *σ *= 10^-5^. In future, we are planning to empirically compare performance with other existing kernel integration formulations on various datasets, and discuss convergence properties of the DC algorithm for MKLdiv-dc based on the theoretical results of [36].

## Authors' contributions

YY and CC conceived the project. YY proposed and implemented the method, drafted the manuscript. KH joined the project and participated in the design of the study. All authors read and improved the manuscript.

## Appendix

### Appendix 1 – Column generation method for SILP

Here we briefly describe the column generation method (see e.g. [40]) for SILP (16) to solve the subproblem (15), i.e.(19)

where , and *S*_0_(*α*) = -2Tr(*α*^⊤ ^*α*). The basic idea is to compute the optimum (*λ*, *γ*) by linear programming for a restricted subset of constraints, and update the constraint subset based on the obtained suboptimal (*λ*, *γ*). More precisely, given restricted constraints {*α*_*p *_: *p *= 1, ..., *P*}, first we find the intermediate solution (*λ*, *γ*) by the following linear programming optimization with *P *linear constraints(20)

This problem is often called the *restricted master problem*. Then, we find the next constraint with the maximum violation for the given intermediate solution (*λ*, *γ*), i.e.(21)

If the optimizer α * of the above equation satisfies  then the current intermediate solution (*λ*, *γ*) is optimal for the optimization (19). Otherwise α* should be added to the restriction set. We repeat the above iteration until convergence which is guaranteed to be globally optimal, see e.g. [14,40]. In a similar fashion to the convergence criterion in [14], the algorithm stops when

For instance, the threshold *ε *is usually chosen to be 5 × 10^-4^.

### Appendix 2 – Sparse formulation of kernel learning based on discriminant analysis

In this appendix we show that kernel learning for regularized discriminant analysis [18] is closely related to sparse regularization. To see this, consider the following algorithm

Using the fact [31] that min , the above equation is identical to(22)

The equivalence between the above algorithm and RKDA kernel learning becomes clear if we formulate its dual problem as follows:

**Theorem 2 ***Let *, **I**_*n *_*be the identity matrix and ***1**_*n *_*be an n-dimensional column vector of all ones. Define *, *and **for any i *∈ ℕ_*n*_. *Then, the dual problem of algorithm (22) can be written as*

where .

**Proof**: Taking the minimization of b first, algorithm (22) yields . Then, algorithm (22) can be further rewritten as(23)

Here, for any ℓ and *i*,  which can be further represented by . Then, letting  for any *i *and solving the standard Lagrangian formulation of (23) with Lagrangian variables *α *yields

Now, replacing *α*_*i *_by *μα*_*i *_and letting  completes the argument. □

Let *n*_- _and *n*_+ _denote the number of samples in class +1 and -1. If we redefine the class indicator output **y**, for any *i *∈ ℕ_*n *_by *y*_*i *_=  if *x*_*i *_is in class +1 otherwise -, then the class indicator output  reduces to the vector *a *defined in [18] for binary classification, i.e.

Now we turn our attention to multiclass classification. To this end, consider

Using the above argument for binary classification it is easy to check its dual problem is as follows(24)

where . Let *n*_*c *_denote the number of samples in class *c*. If we redefine the class indicator matrix **Y**, for any *i *∈ ℕ_*n *_and *c *∈ ℕ_*C *_by  if *y*_*i *_= *c*, otherwise , then the class indicator matrix  reduces to the matrix *H *defined in [18] for multi-class classification, i.e.

Now we can see that the dual problem of algorithm (24) is exactly the same as the formulation (see equation (36) in [18]) of RKDA kernel learning.
